# Guide for library design and bias correction for large-scale transcriptome studies using highly multiplexed RNAseq methods

**DOI:** 10.1186/s12859-019-3017-9

**Published:** 2019-08-13

**Authors:** Shintaro Katayama, Tiina Skoog, Cilla Söderhäll, Elisabet Einarsdottir, Kaarel Krjutškov, Juha Kere

**Affiliations:** 1 Department of Biosciences and Nutrition, Karolinska Institutet, 14183 Huddinge, Sweden; 2 Department of Women’s and Children’s Health, Karolinska Institutet, 17177 Stockholm, Sweden; 3 Folkhälsan Institute of Genetics, and Molecular Neurology Research Program, University of Helsinki, 00014 Helsinki, Finland; 4 Competence Centre on Health Technologies, 50410 Tartu, Estonia; 5 School of Basic and Medical Biosciences, King’s College London, Guy’s Hospital, London, SE1 9RT UK

**Keywords:** Gene expression, Next-generation sequencing, Transcriptome, Library bias correction

## Abstract

**Background:**

Standard RNAseq methods using bulk RNA and recent single-cell RNAseq methods use DNA barcodes to identify samples and cells, and the barcoded cDNAs are pooled into a library pool before high throughput sequencing. In cases of single-cell and low-input RNAseq methods, the library is further amplified by PCR after the pooling. Preparation of hundreds or more samples for a large study often requires multiple library pools. However, sometimes correlation between expression profiles among the libraries is low and batch effect biases make integration of data between library pools difficult.

**Results:**

We investigated 166 technical replicates in 14 RNAseq libraries made using the STRT method. The patterns of the library biases differed by genes, and uneven library yields were associated with library biases. The former bias was corrected using the NBGLM-LBC algorithm, which we present in the current study. The latter bias could not be corrected directly, but could be solved by omitting libraries with particularly low yields. A simulation experiment suggested that the library bias correction using NBGLM-LBC requires a consistent sample layout. The NBGLM-LBC correction method was applied to an expression profile for a cohort study of childhood acute respiratory illness, and the library biases were resolved.

**Conclusions:**

The R source code for the library bias correction named NBGLM-LBC is available at https://shka.github.io/NBGLM-LBC and https://shka.bitbucket.io/NBGLM-LBC. This method is applicable to correct the library biases in various studies that use highly multiplexed sequencing-based profiling methods with a consistent sample layout with samples to be compared (e.g., “cases” and “controls”) equally distributed in each library.

**Electronic supplementary material:**

The online version of this article (10.1186/s12859-019-3017-9) contains supplementary material, which is available to authorized users.

## Background

RNA sequencing (RNAseq) utilizing next generation sequencers has allowed for rapid and comprehensive expression profiling. Due to the reduction of required RNA amount and increasing multiplexity in RNAseq libraries, RNAseq is increasingly used also for large-scale cohort studies based on bulk tissues, as well as tissue complexity studies even at the single-cell scale. However, large-scale studies often require sample processing as multiple libraries, resulting in the risk of batch effect bias. As an example, using our published RNAseq protocol [1], we include up to 48 samples in one library using barcode sequences for multiplexing, processing typically up to four libraries in 3 days, and larger studies often require even more libraries. After sequencing and preprocessing, we sometimes observe a library bias – for example, reference samples in two different libraries may cluster apart by the libraries. Similar issues, especially in studies using single-cell RNAseq methods [2–4], have been recently reported. Therefore, further investigation of the sources of library bias, development of correction methods, and guidelines for a bias-tolerant experiment design are required. In this report, we investigated library biases using 166 technical replicates distributed over 14 libraries. We developed a bias correction method named NBGLM-LBC, and suggest library design guidelines, applicable to many large-scale RNAseq studies.

## Materials and methods

### Preparation of reference RNAs

The present study was based on reference samples in two projects: one performed with BEAS-2B cells, a human bronchial epithelial cell line, and the other with THP-1 cells, a human monocytic cell line. The cells were obtained from the American Type Culture Collection. For RNA extraction, 0.5 × 10^6^ BEAS-2B cells or 1.0 × 10^6^ THP-1 cells were seeded on 12-well plates 48 h/24 h before extraction, respectively. The cells were washed twice with ice-cold Phosphate Buffered Saline (PBS) and lysed with 350 μl of Buffer RLT Plus supplemented with β-mercaptoethanol (AllPrep DNA/RNA/miRNA Universal Kit, Qiagen, Hilden, Germany). The lysates were homogenized and the total RNA was extracted according to manufacturer’s instructions. The quality and quantity of the total RNA samples were checked by NanoDrop (ThermoFisher, Carlsbad, CA) and Qubit (ThermoFisher) instruments. RNA integrity was measured by Bioanalyzer with an Agilent RNA 6000 Nano kit (Agilent Technologies, Santa Clara, CA). The RNA samples were used for sequencing by single-cell tagged reverse transcription (STRT) RNAseq method.

### Preparation and sequencing of STRT libraries

High-quality RNA samples were diluted to 10 ng/μl based on Qubit fluorometer (ThermoFisher) measurements. Illumina-compatible RNA-seq libraries were prepared according to our published STRT protocol [1] using 10 PCR cycles. The ready library QC was performed by TapeStation DNA HS1000 assay (Agilent Technologies) and quantified by qPCR-based KAPA Library Quantification Kit (KK4835, KAPA Biosystems, Cape Town, South Africa). Sequencing was performed using Illumina’s HiSeq 2000 instrument (Illumina, San Diego, CA) according to [1].

### Preprocessing of STRT sequencing results

The sequenced STRT raw reads were processed by STRTprep [1], v3dev branch b866538 commit at https://github.com/shka/STRTprep/tree/v3dev. In brief, the reads were demultiplexed by the barcode sequences, and the best quality reads among redundant reads were selected. The nonredundant reads were aligned to hg19 human reference genome sequences, ERCC spike-in sequences and human ribosomal DNA unit (GenBank: U13369) with RefSeq [5] transcript alignments as a guide of exon junctions, by TopHat2 [6] with bowtie1 [7]. Uniquely mapped reads within (i) the 5′-UTR or the proximal upstream (up to 500 bp) of the RefSeq protein coding genes, and (ii) within the first 50 bp of spike-in sequences, were counted.

### Statistics and figures

All statistics and figures were performed in R (version 3.5.1 on macOS 10.13), and the core source code is provided in Additional file 1. Spike-in normalization was based on the sum of spike-in reads as in [8]. Quantile normalization [9] via the preprocessCore package at https://github.com/bmbolstad/preprocessCore and VST normalization [10] were done after adding 1 to all reads to avoid log_2_(0). The sum of all counts per sample was used as the mapped depth for RPM normalization [11]. Half of the minimum expression level was added to all before log_2_ transformation for spike-in and RPM normalized levels, to avoid log_2_(0). Hierarchical clustering of the Spearman correlation matrix and the corresponding plots were made using a heatmap function in the NMF package [12]; non-log normalized levels with zero-expression masking (by NA) were applied for calculation of the Spearman correlation matrix. Principal component analysis (PCA) and the corresponding plots were produced using the factoextra package at http://www.sthda.com/english/rpkgs/factoextra; log_2_ normalized levels were applied. Genes with biological variation (fluctuated genes) in the GEWAC dataset had a significantly higher gene-to-spike-in ratio in the squared coefficient of variation, described in Supplementary Text S1 of [1]; for the definition of fluctuated genes, see [13].

### Implementation

NBGLM-LBC is developed for correction of library bias in large-scale RNAseq experiments. It is implemented in R, and requires the “MASS” and “parallel” core packages. The source code and documentation are available at https://shka.github.io/NBGLM-LBC under the MIT license; the site is mirrored at https://shka.bitbucket.io/NBGLM-LBC. A read count matrix before normalization (rows are genes, and columns are samples), a vector of sequencing depths, and a factor of libraries are given for the function named “library_bias_correction”. Optionally, multiple CPU cores can be allocated, to speed up the analysis. NBGLM-LBC estimates regression lines between the raw read count and the sequencing depths per library in each gene based on the negative binomial distribution, and then corrects the library biases by making intercepts of the regression lines equivalent based on the assumption that average levels per library are equivalent between libraries; the statistical background of this implementation is explained in the Results section. The output is a corrected count matrix. After the correction, another traditional normalization method will be required for the downstream analysis.

## Results

### Definition and investigation of library bias

The present study was based on reference samples in two projects: one performed with BEAS-2B cells, a human bronchial epithelial cell line, and the other with THP-1 cells, a human monocytic cell line. We used STRT RNAseq [1] for both projects, using 10 ng of total RNA per sample. A reference sample (total RNA from one aliquot of untreated BEAS-2B and THP-1 cells, respectively) for each project was put into 99 wells over eight STRT libraries for BEAS-2B, and 67 wells over six libraries for THP-1 as technical replicates (out of 360 and 271 samples, respectively; Additional file 2); there were samples with specific treatments and their controls in the other wells. As with most highly multiplexed RNAseq methods, these samples were barcoded and amplified per sample, followed by pooling and amplification per library. In one experiment, a maximum of four parallel libraries were made by the same experienced laboratory engineer, and all libraries were sequenced to the same depth, three Illumina HiSeq 2000 lanes per library (Table 1). After preprocessing to deconvolute the samples to raw sequences, there were at least one million total reads for all the replicates, and there was no significant RNA degradation in any sample (Additional file 2). The processed raw reads (Additional files 3 and 4) were used for the downstream analysis.

**Table 1:** The history of library preparation and sequencing for investigation of the library biases

Library		Library synthesis (date)			Sequencing (flowcell:lane)			
Project	ID	RNA into capture plate	Started	Finished	Quantity (nM)	1st	2nd	3rd
BEAS	a	2015/12/30	2016/01/04	2016/01/07	0.50	A:6	B:1	C:1
BEAS	b	2015/12/30	2016/01/04	2016/01/07	0.26	A:7	B:2	C:2
BEAS	c	2015/12/30	2016/01/04	2016/01/07	1.71	A:8	B:3	C:3
BEAS	d	2015/12/30	2016/01/04	2016/01/07	0.82	B:4	C:4	D:5
BEAS	e	2015/12/30	2016/01/08	2016/01/11	1.11	B:5	C:5	D:6
BEAS	f	2015/12/30	2016/01/08	2016/01/11	1.10	B:6	C:6	D:7
BEAS	g	2015/12/30	2016/01/08	2016/01/11	1.01	B:7	C:7	D:8
BEAS	h	2015/12/30	2016/01/08	2016/01/11	1.40	B:8	C:8	E:1
THP1	a	2015/12/04	2015/12/07	2015/12/09	1.50	A:1	F:3	G:2
THP1	b	2015/12/04	2015/12/07	2015/12/09	2.10	A:2	F:4	G:3
THP1	c	2015/12/04	2015/12/08	2015/12/10	2.00	F:5	G:4	G:5
THP1	d	2015/12/04	2015/12/08	2015/12/10	1.90	A:3	F:6	G:6
THP1	e	2015/12/07	2015/12/08	2015/12/10	2.90	A:4	F:7	G:7
THP1	f	2015/12/07	2015/12/08	2015/12/10	2.70	A:5	F:8	G:8

**Fig. 1:**
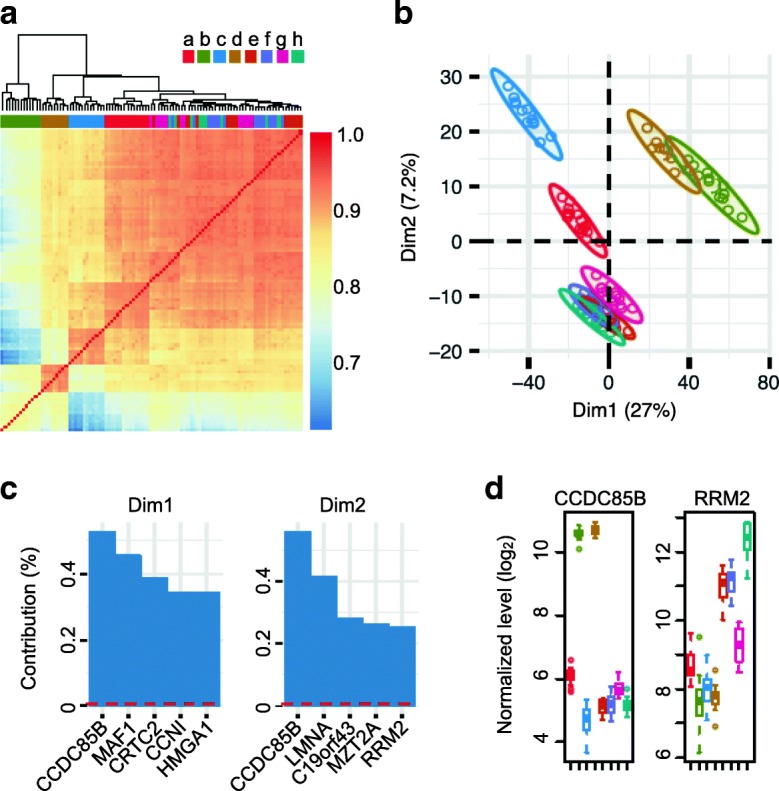
Biases between BEAS-2B libraries. **a** Spearman correlation coefficients between the 99 technical replicates over 8 libraries and the similarities; the color scheme for representation of the libraries is common also for the following panels. **b** PCA on VST [10] normalized expression levels of the replicates. **c** The top 5 contributing genes to the dimensions. Red dashed lines correspond to the expected value if the contributions are uniform. **d** The normalized levels of *CCDC85B* and *RRM2*

Although the reference samples were technical replicates of the same RNA, hierarchical clustering as well as PCA grouped the samples by the libraries (Fig. 1a and b). This is the “library bias” that we are investigating in this report. The library bias remained despite using spike-in [8], quantile [9], RPM [11] or VST [10] normalization methods (Additional file 5: Figure S1 & S2). Amplification bias should be independent of the library bias, as we are using UMIs [14] in this STRT protocol. In the BEAS-2B libraries, *CCDC85B* and *RRM2* were the most biased genes because of their high contribution to the principal components (Fig. 1c). The biases between libraries were more than 16-fold, and the patterns were different between the biased genes (Fig. 1d; *CCDC85B* was high in BEASb and d libraries, while *RRM2* was high in BEASh), but the patterns were conserved in the normalization methods (Additional file 5: Figure S3). Therefore, an additional correction should be applied for each gene before the normalization.

### Implementation of a library bias correction method, NBGLM-LBC

**Fig. 2:**
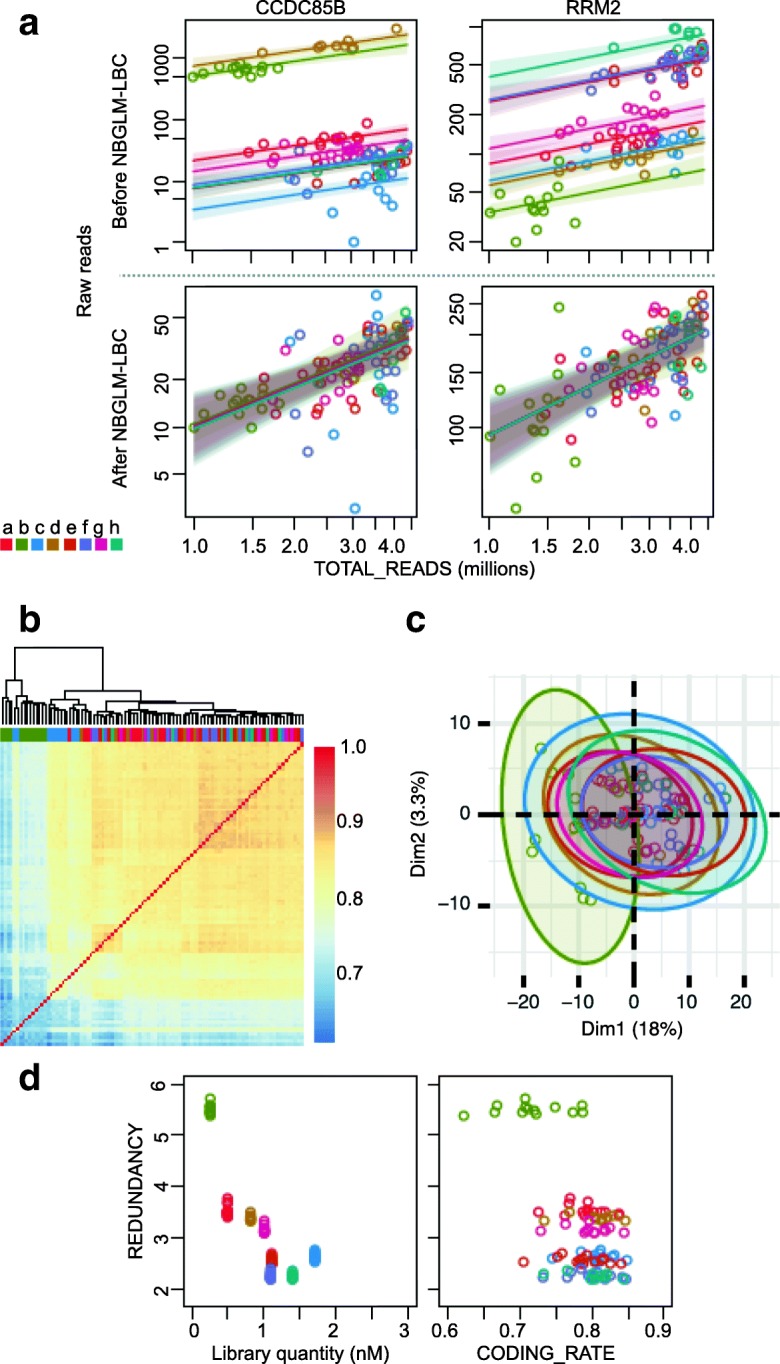
Library bias correction to the BEAS-2B libraries. **a** Correlation between the sequencing depths (x-axis) and the raw read counts (y-axis) of *CCDC85B* (left) and *RRM2* (right) before (top) and after NBGLM-LBC (bottom); solid lines were the linear regressions, and the transparent bands were the 95% prediction intervals. **b** Spearman correlation coefficients between the replicates after NBGLM-LBC. **c** PCA on VST normalized expression levels after the library bias correction. **d** Relation between the library quantity (x-axis of the left panel), proportions of mapped reads on protein coding genes (x-axis of the right panel), and the library redundancy (y-axis)

While investigating possible correction methods, we found that the raw read counts and the sequencing depth were correlated in each library, but the regressions differed between the libraries (Fig. 2a, top). Therefore, the library bias of each gene can be reduced by (i) calculating a linear approximation between the depths and the raw counts in each library, and (ii) transforming the raw counts to approximations which overlap each other (Fig. 2a, bottom). Based on this approach, a library bias correction method using a generalized linear model of the negative binomial family with a logarithmic link, named NBGLM-LBC, was implemented. After the library bias correction (Additional files 6 & 7), overall correlation coefficients between the replicates were much improved (Additional file 5: Figure S4) for both reference types (Additional file 5: Figure S5), and the replicates were more similar (Additional file 5: Figure S6) in the four normalization methods (Additional file 5: Figure S7). However, even after correction, the BEASb library tended to be an outlier (Fig. 2b and c). This library was characterized by (i) the lowest number of processed reads (TOTAL_READS in Additional file 2) due to the highest sequence redundancy according to the UMIs (REDUNDANCY in Fig. 2d and Additional file 2), (ii) the lowest library quantity (Fig. 2d and Table 1; the quantity tends to negatively correlate to the redundancy), and (iii) the lowest mapping rate to protein coding genes (CODING_RATE in Fig. 2d and Additional file 2), suggesting low performance of the reverse transcriptase, or high contamination of reads derived from non-mRNAs, during BEASb library synthesis. In contrast, library biases between all THP-1 libraries were well corrected (Additional file 5: Figure S6 bottom). The THP-1 library quantities were very similar, supporting their importance for the effectiveness of the correction (Additional file 5: Figure S8 bottom). In summary, although it was difficult to recover libraries with problems in the library synthesis, NBGLM-LBC reduced the library biases which we observed in technical replicates of the large experiments.

### Simulations suggested that consistent sample layout is required for NBGLM-LBC

**Fig. 3:**
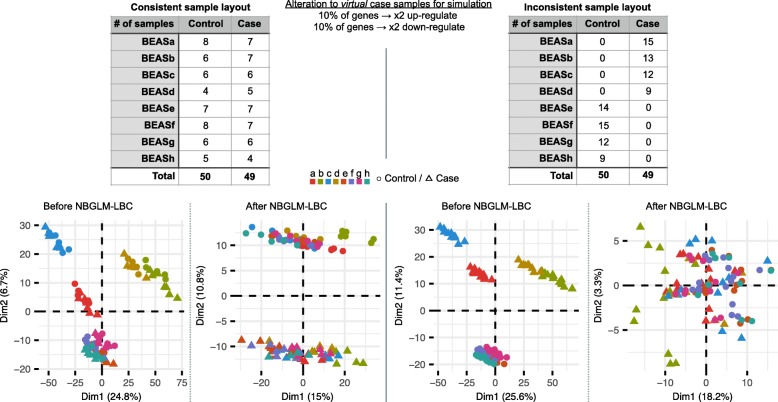
NBGLM-LBC required consistent sample layout. Tables (top) are sample layouts of two simulation datasets. A consistent sample layout (left) is defined as the number of control samples and case samples in each library being almost the same, while in an inconsistent sample layout (right) each library has either control or case samples. Panels (bottom) are PCAs on VST normalized expression levels before (left in each sample layout) and after (right in each sample layout) NBGLM-LBC

In actual studies, we typically compare groups of samples to find differences between them. To evaluate whether NBGLM-LBC is applicable to more common study designs, we tested the method on artificially perturbed expression profiles. The artificial profiles were made for the BEAS-2B libraries by choosing half of the samples as “case” samples. The raw counts of 10% of genes in the case samples were increased twofold, and another 10% of the genes were decreased twofold (Fig. 3 top). When we set half of the samples in each library as “case” samples and half as “controls” (hereafter called “consistent sample layout”), PCA after NBGLM-LBC showed evident differences between control and the case samples (Fig. 3 left). This is expected, as the fold differences between the cases and the controls in each library are conserved – NBGLM-LBC finds regression lines between the cases and the controls in each library, and applies the same transformation to both sample types in same library. On the contrary, when each library only has either “case” samples or “controls” (here called “inconsistent sample layout”), NBGLM-LBC neutralized the differences between the cases and the controls (Fig. 3 right). This is due to NBGLM-LBC transforming regression lines of the case and control libraries so they overlap. The simulation thus suggests that NBGLM-LBC requires a sample layout where both cases and controls are equally present in each library.

### Application case – NBGLM-LBC for a large-scale leukocyte expression profile

**Fig. 4:**
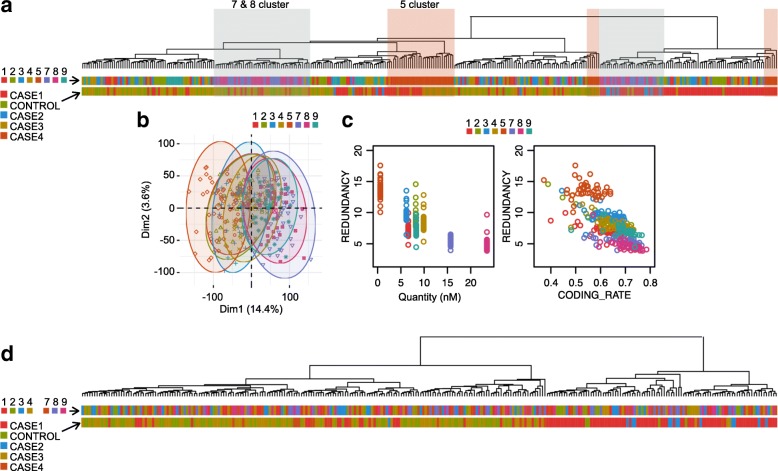
Library biases and the correction in GEWAC study. **a** Hierarchical clustering of GEWAC subjects using the leukocyte expression profile without the library bias correction. Upper bar below tree represents the libraries, and lower bar represents the sample types. **b** PCA of the expression profile without the library bias correction. **c** Relation between the library quantity (x-axis of the left panel), proportions of mapped reads on protein coding genes (x-axis of the right panel), and the library redundancy (y-axis). **d** Hierarchical clustering of GEWAC subjects using the leukocyte expression profile with the library bias correction. The upper bar below tree represents the libraries, the lower bar represents the sample types

GEWAC (Gene Expression in Wheezing and Asthmatic Children) is a study of childhood acute respiratory illness [15, 16]. A leukocyte expression profile using STRT RNAseq [1] with 80 ng of total RNAs and GlobinLock [17] was performed in patients and healthy volunteers. We arranged the same numbers of the controls and the cases in all eight libraries as much as possible, to obtain a consistent sample layout. In unsupervised hierarchical clustering of 2516 fluctuated protein coding genes, library 5 and libraries 7 + 8 tended to cluster together (Fig. 4a). The library biases were observed also in PCA (Fig. 4b). The quantity of the libraries and the CODING_RATE were exceptionally low in library 5, and the redundancy of library 5 was higher than the others (Fig. 4c). We therefore decided to exclude library 5, and applied NBGLM-LBC to the raw counts before normalization and the downstream analysis. Although the major variation between CASE1 samples and the others (at the first branch) was still evident even after the correction, clusters of the library 5 and the libraries 7 + 8 disappeared (Fig. 4d). Therefore, down-stream analysis using the corrected expression profile is less influenced by the library biases. For example, to detect correlating gene modules, and to relate the modules to clinical traits, the biases must be minimized before WGCNA [18].

## Conclusions

The NBGLM-LBC method described here can correct library biases which cannot be corrected by more traditional normalization methods. A consistent sample layout for all libraries in a study is required for the library bias correction by NBGLM-LBC. This is similar to “a balanced study design” proposed by Tung and collaborators [4]. Several library-bias tolerant statistics have been reported previously, such as SAM [19] with block permutations as an example of a bias-tolerant differential expression test. However, the proposed correction method enables various traditional statistical approaches, e.g., hierarchical clustering, PCA, WGCNA or unsupervised clustering of scRNAseq data (reviewed in [20]). Moreover, we can test differential expression using the corrected read counts (but without normalization) by various test methods, e.g., DESeq [10] or SAMstrt [8].

The NBGLM-LBC is potentially applicable also to other studies using highly multiplexed RNAseq methods for single-cells or low-input samples by equivolume pooling of barcoded cDNAs and amplification of the pool. Moreover, it may also be worth applying NBGLM-LBC to other sequencing-based profiling methods (e.g. short-RNAseq or ChIPseq), where significant library biases may be seen. Studies based on lower multiplexity (e.g. 6-plex) or equimolar pooling as performed in several large-input RNAseq protocols, are less likely to benefit from NBGLM-LBC, due to the smaller number of samples per library, or lower variation of the sequence depth between the samples. UMIs are useful for reducing amplification bias [14], but are alone likely not sufficient to reduce library bias. Redundancy, which can be estimated by UMIs, was associated with the library quality, but we can find the redundancy only after sequencing. Therefore, one practical thing to highlight is that measurement of library quantity before sequencing is an important checkpoint. Exceptionally low-quantity libraries in a study should be discarded even if they initially appear to be adequate for sequencing, as the sequence data quality is likely to be insufficient for the expression analysis.

## Availability and requirements

Project name: NBGLM-LBC.

Project home page: https://shka.github.io/NBGLM-LBC (mirrored at https://shka.bitbucket.io/NBGLM-LBC).

Operating system: Platforms supporting the fork system call (ex. Linux or macOS).

Programming language: R (version 3.0 or higher).

License: MIT.

Any restrictions to use by non-academics: No.

## Additional files

Additional file 1: Sample script. This script demonstrates the library bias correction by NBGLM-LBC, performs the simulation experiments for Fig. 3, and draws Figs. 1b, 2a, c and 3 bottoms. Before running this script using R, LBC-additionalFile2.txt (Additional file 2) and LBC-additionalFile3.txt (Additional file 3) must be placed within the working directory, and the preprocessCore, DESeq2 and factoextra packages must be installed. (R 10 kb)

Additional file 2: Layout of reference samples and the qualities. There are seven quality measures provided in the STRTprep pipeline; (i) TOTAL_READS is a read count after the redundant read exclusion based on UMI; (ii) REDUNDANCY is redundancy based on UMI; (iii) MAPPED_RATE is the rate of mapped reads; (iv) SPIKEIN_5END_RATE is the 5′-end capture rate of spike-in molecules; (v) CODING_5END_RATE is the 5′-end capture rate of protein coding genes; (vi) CODING_READS is reads that were aligned to exons of protein coding genes; and (vii) CODING_RATE is calculated by CODING_READS/(TOTAL_READS*MAPPED_RATE). See also detailed instruction at https://github.com/shka/STRTprep/blob/v3dev/doc/result.md#outbygenesamples_allcsv. (TXT 16 kb)

Additional file 3: Raw read counts of BEAS-2B libraries before the library bias correction (TXT 3580 kb)

Additional file 4: Raw read counts of THP-1 libraries before the library bias correction (TXT 2541 kb)

Additional file 5: **Figure S1.** Spearman correlation coefficients between the technical replicates of normalized expression levels by four different normalization methods and the libraries. **Figure S2.** PCA of the BEAS-2B technical replicates of normalized expression levels by four different normalization methods. **Figure S3.** Normalized expression levels by the four different normalization methods, and the ranks, of three library-biased genes. **Figure S4.** Pairwise comparison of raw read counts and the Spearman correlation coefficients before and after the library bias correction. **Figure S5.** Spearman correlation coefficients between the technical replicates of the normalized expression levels before and after the library bias correction. **Figure S6.** PCA of the technical replicates of the spike-in normalized expression levels before and after the library bias correction. **Figure S7.** PCA of normalized expression levels of the BEAS-2B technical replicates by four different normalization methods, before and after the library bias correction. **Figure S8.** Quantity of the library before sequencing, and REDUNDANCY and CODING_RATE after the sequencing. (PDF 2469 kb)

Additional file 6: Raw read counts of BEAS-2B libraries after the library bias correction (TXT 3574 kb)

Additional file 7: Raw read counts of THP-1 libraries after the library bias correction (TXT 2536 kb)

## Data Availability

The source code, the documentation and sample code for NBGLM-LBC are included in the project home page and Additional file 1. The count data before and after the library bias correction (for Table 1, Figs. 1, 2, 3, Additional files 2 and 5) are in Additional files 3, 4, 6 and 7.

## Abbreviations

GEWAC: Gene Expression in Wheezing and Asthmatic Children
PCA: Principal component analysis
STRT: Single-cell tagged reverse transcription
UMI: Unique molecular identifier
