# Supplementary material for: Guide for library design and bias correction for large-scale transcriptome studies using highly multiplexed RNAseq methods
Source: BMC Bioinformatics. 2019 Aug 13;20:418. doi: 10.1186/s12859-019-3017-9 (PMC6693229; doi:10.1186/s12859-019-3017-9)
Supplement: Supplementary file 5 — Figure S1. Spearman correlation coefficients between the technical replicates of normalized expression levels by four different normalization methods and the libraries. Figure S2. PCA of the BEAS-2B technical replicates of normalized expression levels by four different normalization methods. Figure S3. Normalized expression levels by the four different normalization methods, and the ranks, of three library-biased genes. Figure S4. Pairwise comparison of raw read counts and the Spearman correlation coefficients before and after the library bias correction. Figure S5. Spearman correlation coefficients between the technical replicates of the normalized expression levels before and after the library bias correction. Figure S6. PCA of the technical replicates of the spike-in normalized expression levels before and after the library bias correction. Figure S7. PCA of normalized expression levels of the BEAS-2B technical replicates by four different normalization methods, before and after the library bias correction. Figure S8. Quantity of the library before sequencing, and REDUNDANCY and CODING_RATE after the sequencing. (PDF 2469 kb) [file 12859_2019_3017_MOESM5_ESM.pdf]

Supplementary Figures

Katayama *et al.* Guide for library design and bias correction for large-scale transcriptome studies using highly multiplexed RNAseq methods

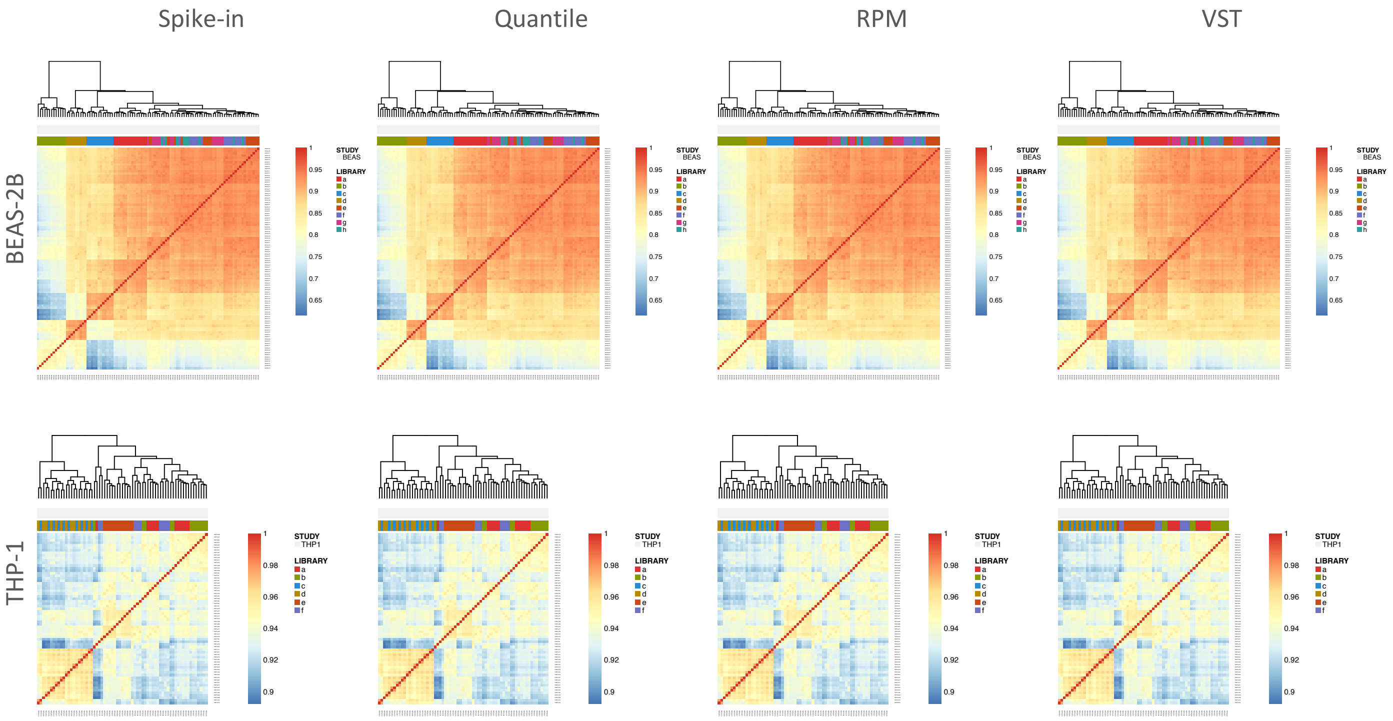

Figure S1. Spearman correlation coefficients between the technical replicates of normalized expression levels by four different normalization methods and the libraries.

The reference samples should show weak clustering, without library dependency, as they were technical replicates of the same RNA sample. However, there were some significant clusters, especially BEASb. All normalization methods transform the average and/or the variation of expression levels in each sample but do not change the ranks in each sample. Therefore, the library biases, which were represented by distances of Spearman rank correlation coefficients here, were identical between the methods.

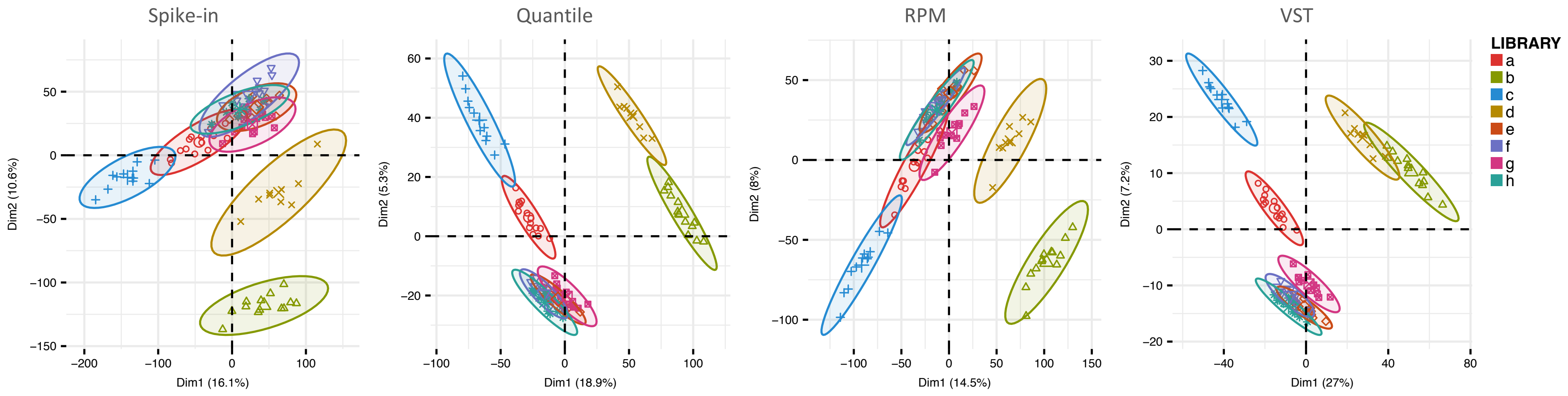

Figure S2. PCA of the BEAS-2B technical replicates of normalized expression levels by four different normalization methods.

Considering that these samples are technical replicates, they should be clustered without library dependency. Instead, they showed clustering by libraries.

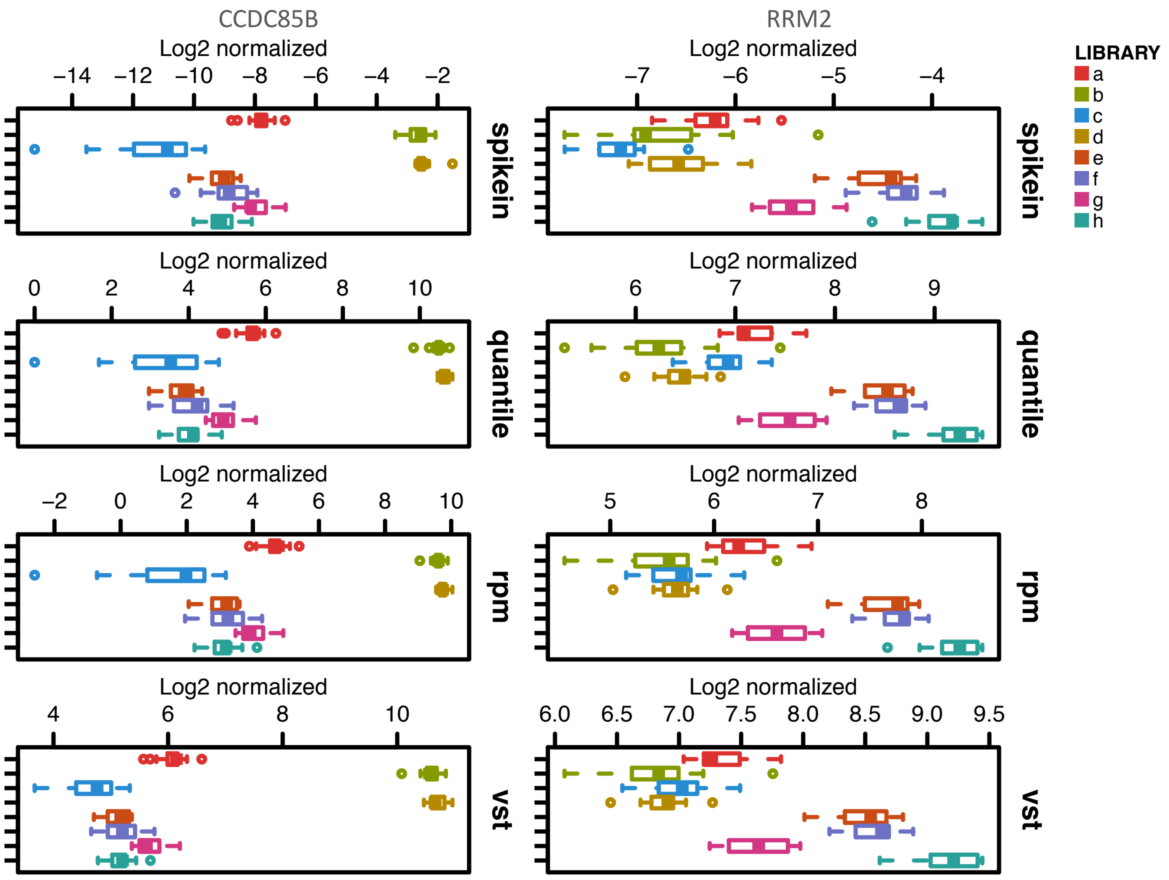

Figure S3. Normalized expression levels by the four different normalization methods of two library-biased genes.

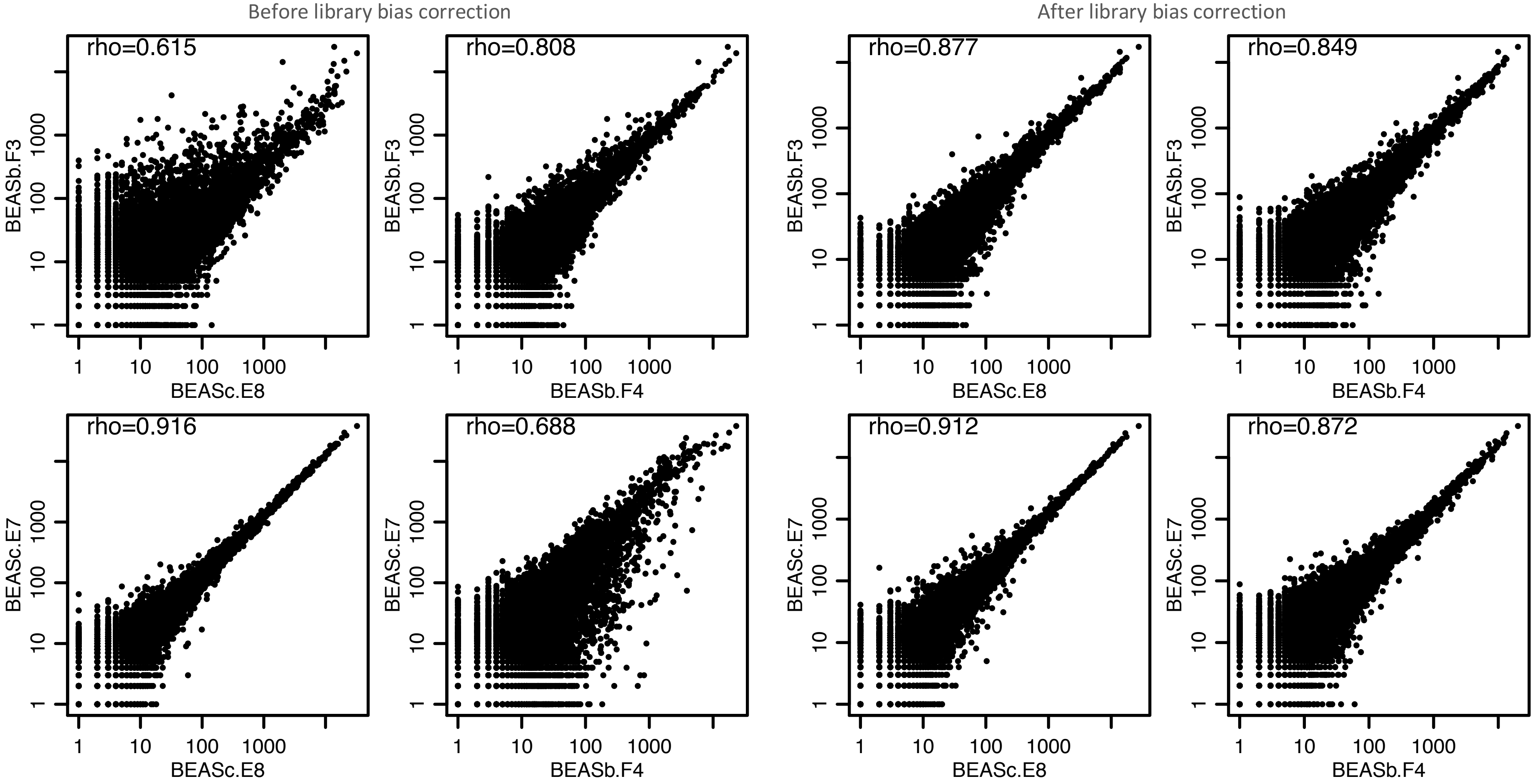

Figure S4. Pairwise comparison of raw read counts and the Spearman correlation coefficients before and after the library bias correction.

BEASb.F3 and BEASc.E8 was the worst reference sample pair before the library bias correction.

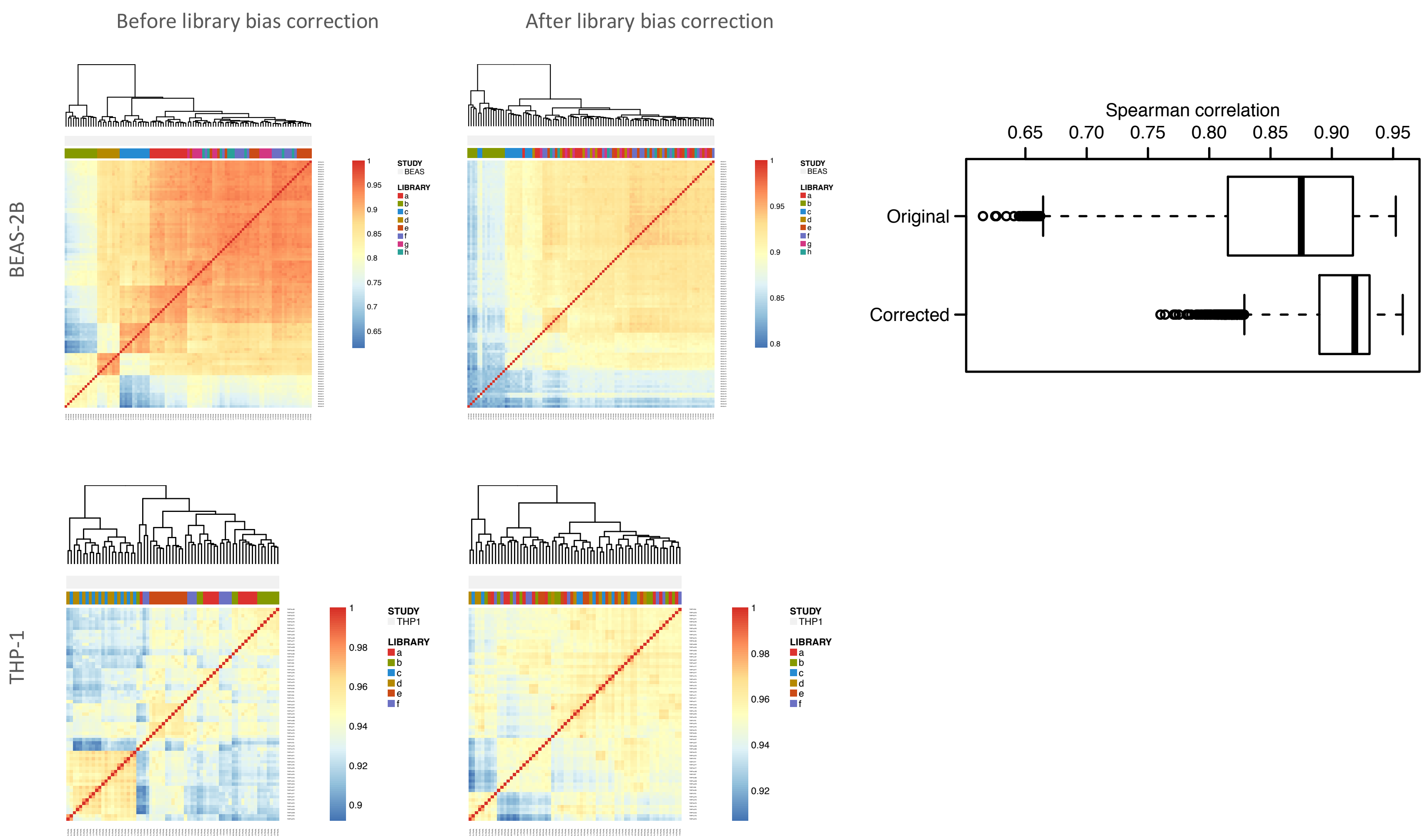

Figure S5. Spearman correlation coefficients between the technical replicates of the normalized expression levels before and after the library bias correction.

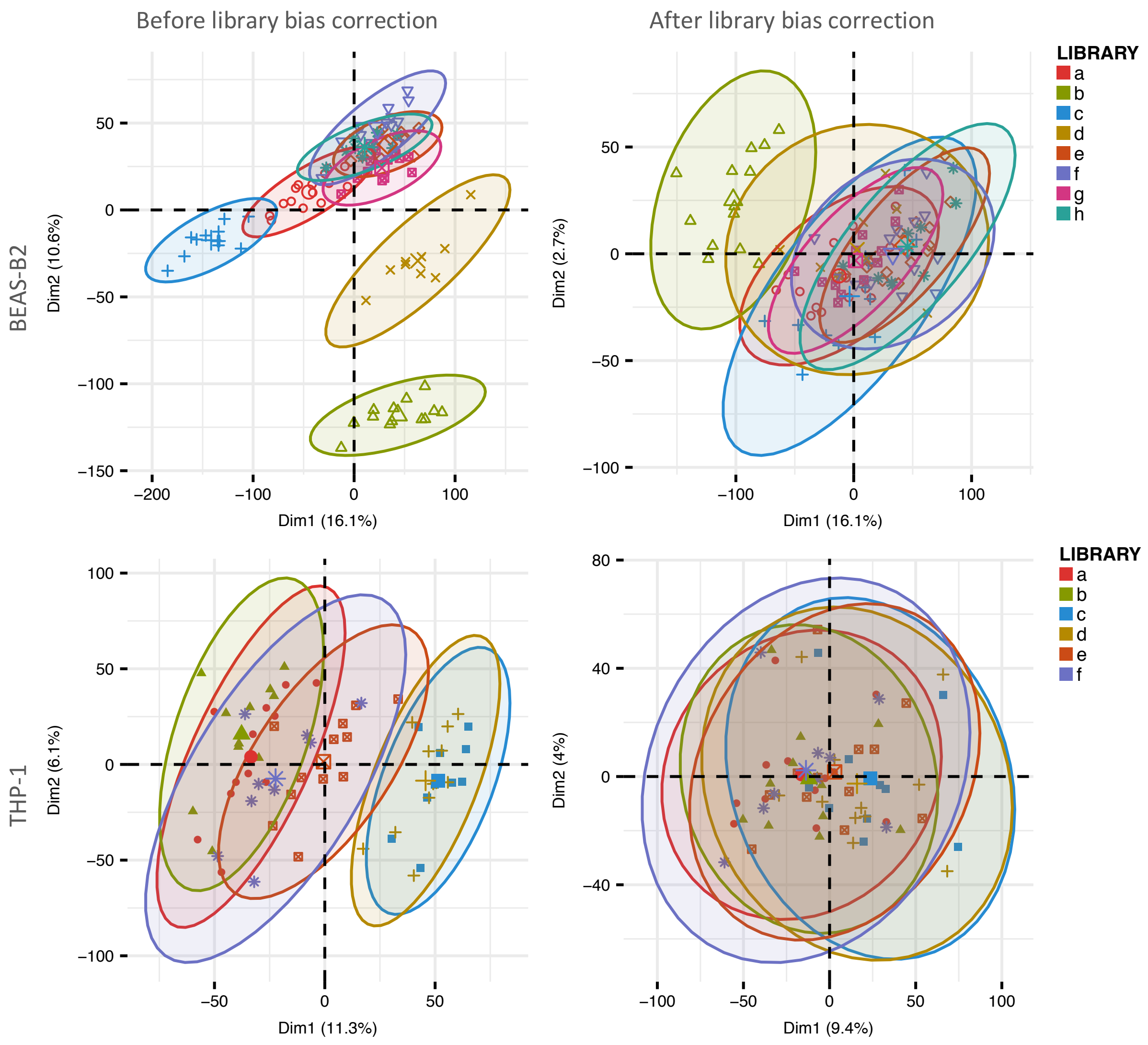

Figure S6. PCA of the technical replicates of the spike-in normalized expression levels before and after the library bias correction.

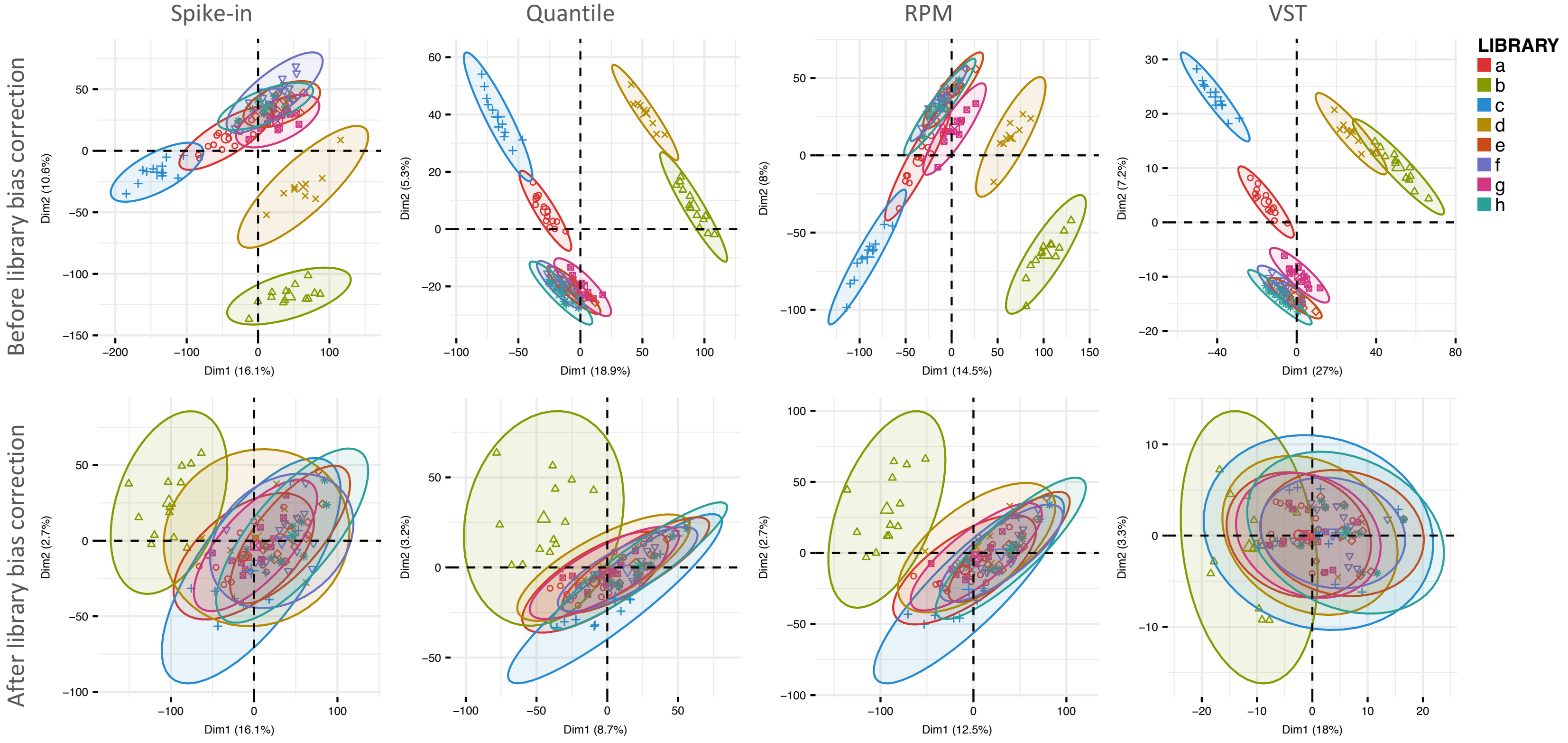

Figure S7. PCA of normalized expression levels of the BEAS-2B technical replicates by four different normalization methods, before and after the library bias correction.

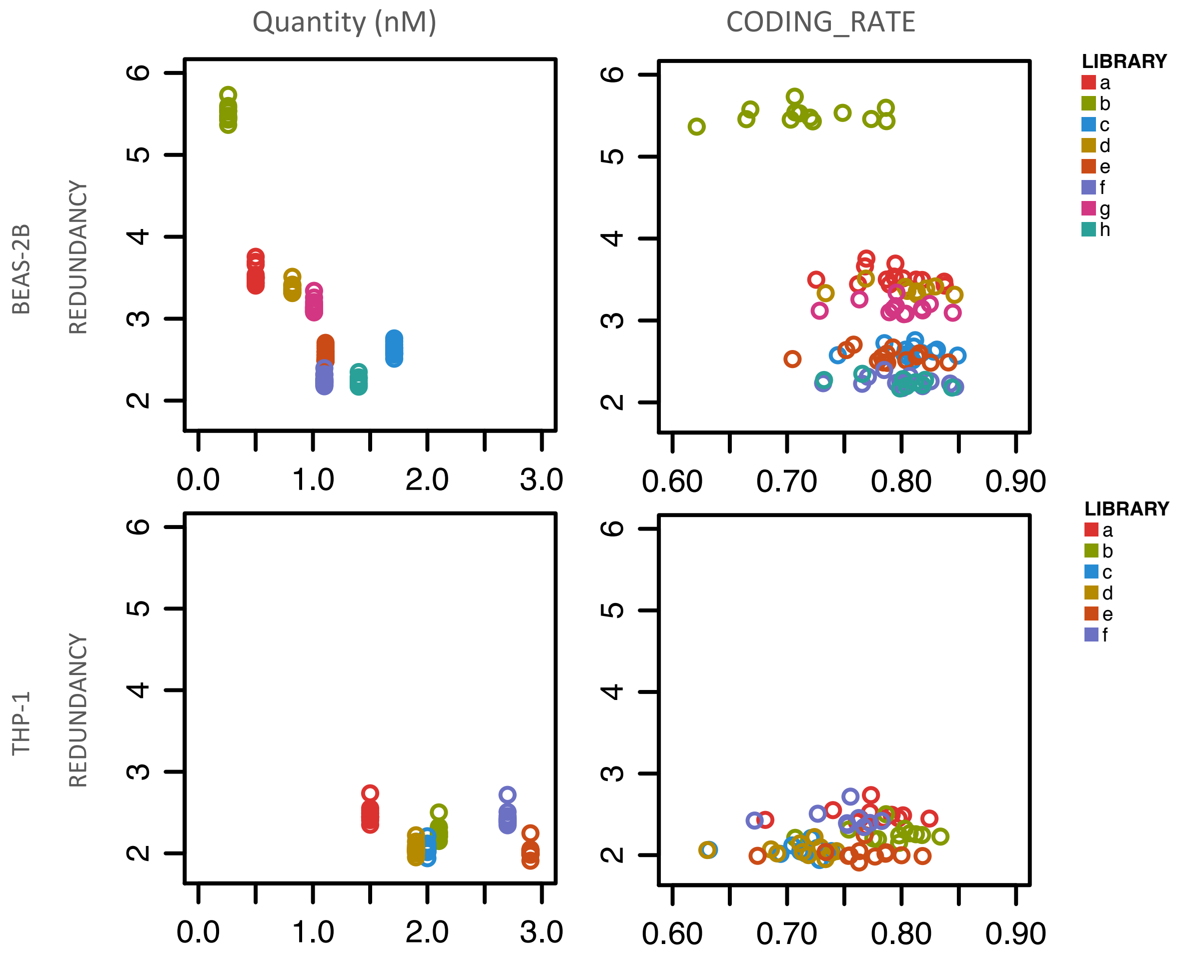

Figure S8. Quantity of the library before sequencing, and REDUNDANCY and CODING_RATE after the sequencing.

Drop of the quantity in the libraries using similar samples in a study suggests potential technical issues. (NOTE: REDUNDANCY and CODING_RATE are different by types of cells or tissues as well as the amount of input RNAs
